# Use of melatonin in oral health and as dental premedication

**DOI:** 10.1186/s40709-015-0036-1

**Published:** 2015-11-19

**Authors:** Mercedes Perez-Heredia, Javier Clavero-González, Leticia Marchena-Rodríguez

**Affiliations:** School of Dentistry, University of Granada, Campus Universitario de Cartuja s/n, Granada, Spain, E-18071 Granada, Spain

**Keywords:** Melatonin, Dental, Anxiety, Premedication

## Abstract

Anxiety is a common problem in dentistry which could affect the correct treatment and involve failure. Oral premedication is needed to treat several anxious patients. Many people are so highly anxious that oral sedation is not effective and need to be under deep sedation or even general anaesthesia in order to receive dental care. In these patients, due to a high level of anxiety, even the insertion of an intravenous catheter can be difficult. Benzodiazepines have been the most commonly used anxiolytic in these cases, but many may be associated with paradoxical reactions. Melatonin has a good potential to be used in this field as alternative to benzodiazepines because it may induce a natural sleepiness and improve sedation. The purpose of this paper was to summarize what is known about the use of melatonin in oral health and as dental premedication in anxious dental patients. Databases were searched for the relevant published literature to 30 April 2015. The following search items were used in various combinations: melatonin, premedication, anxiety, dental, sedation and anaesthesia. Few articles were found about this aspect, and the use of melatonin is still a controversial aspect in dental field. More detailed/specific studies are necessary to extend the therapeutic possibilities of melatonin as premedication in dentistry.

## Background

There is evidence that phobias, anxiety or dentist fear may be related to general health problems. This is an important fact if it is considered that more than half of patients need to go to the dentist and 20 % avoid it for that reason. Dentist’s fear tend to be stable and difficult to remove due to the personal experience or anxiety induced by feedback from other patients [1]. In highly anxious patients, if the treatment is provided under general anaesthesia, fear of needles can hamper the placement of an intravenous line or there may be fear of being under anaesthesia in addition to the fear owing to the dental procedure.

There are different methods of helping patients to reduce this sense of fear and anxiety and one of the most effective is oral premedication. The most commonly used oral anxiolytic medication for patients in dentistry has been a drug from the benzodiazepines class, such as triazolam, midazolam, lorazepam, and diazepam [2]. There are widely known side effects of benzodiazepines class, and rejection on several occasions to take the medication. In this sense, substances such as melatonin has showed to be as good in anxiolysis as benzodiazepines but without most of their side effects [3, 4].

## Review

### Biology of melatonin and application in oral health

Melatonin (MT) is the principal secretory product of the pineal gland and associated with the regulation of the circadian dark/light rhythm of the human body. This hormone has antioxidant and inmunomodulatory activities. Additionally, MT does not have any toxicity but is an highly lipophilic substance and this property facilitates its penetration through cell membranes and compartments [5] which suggests that MT could be used therapeutically; for instance, locally, (1) in the oral cavity damage of mechanical, bacterial, viral and fungal origin, (2) in postsurgical wounds caused by tooth extractions and other oral surgeries, acting as a promoter of bone formation, suppressing inflammation of the gingiva and periodontum, (3) to enhance osteointegration of dental implants and in auto-immunological disorders such as Sjorgen syndrome, herpes lesions, aphthous ulceration, lichen planus and (4) even to limit oral cancer [6]. In this sense, the beneficial effects of MT in oral health could be several such as the reduction of severity of herpes, that have proven to be at least as effective as drug Acyclovir, and although it is unclear mechanism of action, it could be due to stimulation of NK CD4 cells [7]. Melatonin in oral health could be considered as a prognostic marker for oral pathologies like cancer. Low plasma levels of MT is associated to oral cancer, while MT could be considered as an oncostatic agent of several types of cancer: such as lung cancer, breast, gastric, colon which are linked by low plasma levels of MT, too [7].

MT gets to the saliva by passive diffusion from the blood. This is why the concentration of MT in saliva was reported to be about 30 % of that in the plasma [8]. The amount of MT in saliva is lower when compared to blood value probably due to the fact that nearly 70 % of the plasma MT is bound to albumin and this prevents its free diffusion into saliva.

Local MT administration reports a lot of potential effects in the oral cavity [9] and may contribute to the regeneration of alveolar bone through the stimulation of type I collagen fiber production and modulation of osteoblastic and osteoclastic activity.

It should be considered that MT from the blood into the saliva may play an important role in suppressing oral diseases and have beneficial effects in periodontal disease, herpes and oral cancer [10] and the importance of MT in the prognosis and treatment of certain tumours, like epidermoid carcinoma [7].

### Premedication in dentistry

Oral sedation can be beneficial in dentistry due to the fact that it decreases activity, moderates excitement, and calms the patient. While barbiturates were effective at sedation, they had certain undesirable side effects, such as potential for addiction, cardiovascular and respiratory depression, and a low therapeutic index. Barbiturate use was eventually replaced by benzodiazepines for sedation due to their wide margin of safety and effectiveness in sedation, anxiolysis, and amnesia. Furthermore, additional care must be taken in patients with cardiovascular, respiratory, renal or hepatic disease [11].

The use of melatonin sublingually (0.05 mg kg^−1^ or 5 mg) was shown to be associated with preoperative anxiolysis in adults without psychomotor impairment or impact on recovery. The melatonin has other advantages such as: (1) it is difficult to overdose, since it is a naturally occurring hormone, (2) it may also be more acceptable by patients that may be uncomfortable intaking synthetic medications and (3) it has a relatively short half-life, so prolonged sedation is less likely than with benzodiazepines [4, 12].

Use of MT is generally safe without significant adverse effect in healthy adults over the age of 18 years old, who are not pregnant and without psychiatric disorders, and the only one short-term side effect from oral administration of exogenous melatonin is sleepiness, but consequences of a long-term melatonin use are still unknown.

### Exogenous melatonin and its use in dentirsty

The way that MT exerts its anaesthetic effect is similar to other anaesthetics such as propofol and benzodiazepines, increasing the binding of GABA to the GABAa receptor, because binding of melatonin to the MT1 receptor appears to affect the GABAa receptor via the G-coupled protein pathway [13].

Exogenous melatonin is rapidly absorbed and peak plasma levels are reached in 60–150 min. The elimination half-life of melatonin is about 12–48 min. Melatonin has a hypnotic/sedative effect when administered orally [3]. This may be due to its circadian rhythm regulation effect. Sublingual administration of melatonin prior to general anaesthesia produce sedation and anxiolysis comparable to sublingual midazolam in adult women, which is significantly different compared to placebo [13].

Melatonin premedication, in an oral dose of either 3 or 5 mg, reduce the required dose of propofol to achieve a bispectral index score 45, reflecting a sufficient level of hypnosis for tracheal intubation without prolongation of postoperative recovery room stay [14] and is associated with faster recovery times and less incidence of postoperative excitement and sleep disturbances 2 weeks postoperatively in comparison with midazolam [15].

In a recently published review to assess the effect of melatonin on pre- and postoperative anxiety in adults, when compared MT given as premedication to placebo, melatonin can reduce preoperative anxiety 50–100 min after administration and MT may be equally as effective as standard treatment with midazolam in reducing preoperative anxiety (50–100 min after administration) in adults [16]. Unlike midazolam, oral melatonin does not impair the general cognitive and psychomotor functions [17].

Melatonin may reduce postoperative anxiety in adults 6 hours after surgery compared to placebo, but its effect is mixed and an overall attenuation of the effect compared to preoperatively is suggested [16]. Oral MT seems prevent postoperative agitation in children who need general anaesthesia for oesophageal dilatation procedures [18].

A randomized controlled factorial trial suggested that adition of MT to alprazolam produces superior anxiolysis compared with either drugs alone or placebo [19].

There is very little investigation on melatonin to reduce anxiety for dental treatment. Only one paper showed similar results when MT is used as dental premedication in children compared with placebo [20]. The possible reason is that perhaps melatonin cannot induce hypnosis/sedation at a high enough level in very anxious dental patients to accept its use as an universal oral premedication. However, comparing MT with other substances such as benzodiazepines (the current gold standard), it seems that melatonin has good potential to be used for reducing anxiety and producing sedation in dental patients undergoing general anaesthesia without any significant adverse effect.

Concluding, further experimental studies are needed about functions of melatonin in the oral cavity. MT can be effective provided it is used in a right population and situation but more specific/detailed studies are necessary to extend the therapeutic possibilities of melatonin as premedication in dentistry.
